# Direct Innominate Artery Cannulation versus Side Graft for Selective Antegrade Cerebral Perfusion during Aortic Hemiarch Replacement

**DOI:** 10.1055/s-0042-1744136

**Published:** 2022-05-31

**Authors:** Anna K. Gergen, Cenea Kemp, Christian V. Ghincea, Zihan Feng, Yuki Ikeno, Muhammad Aftab, T. Brett Reece

**Affiliations:** 1 Division of Cardiothoracic Surgery, Department of Surgery, University of Colorado School of Medicine, Aurora, Colorado

**Keywords:** hemiarch replacement, selective antegrade cerebral perfusion, hypothermic circulatory arrest, direct cannulation, side graft

## Abstract

**Background** Selective antegrade cerebral perfusion (SACP) has become our preferred method for cerebral protection during open arch cases. While the initial approach involved sewing a graft to the innominate artery as the arterial cannulation site, our access strategy has since evolved to central aortic cannulation with use of a percutaneous cannula in the innominate for SACP. We hypothesized that SACP delivered via direct innominate cannulation using a 12- or 14-Fr cannula results in equivalent outcomes to cases utilizing a side graft.

**Methods** This was a single-center, retrospective analysis of 211 adult patients who underwent elective hemiarch replacement using hypothermic circulatory arrest with SACP via the innominate artery between 2012 and 2020. Urgent and emergent cases were excluded.

**Results** A side graft sutured to the innominate was utilized in 81% ( *n* = 171) of patients, while direct innominate artery cannulation was performed in 19% ( *n* = 40) of patients. Baseline patient characteristics were similar between groups aside from a higher baseline creatinine in the direct cannulation group (1.3 vs. 0.9, *p* = 0.032). Patients undergoing direct cannulation demonstrated shorter cardiopulmonary bypass time (132.7 vs. 154.9 minutes, *p* = 0.020) and shorter circulatory arrest time (8.1 vs. 10.9 minutes, *p* = 0.004). Nadir bladder temperature did not significantly differ between groups (27.2°C for side graft vs. 27.6°C for direct cannulation, *p* = 0.088). There were no significant differences in postoperative outcomes.

**Conclusion** Direct cannulation of the innominate artery with a 12- or 14-Fr cannula for SACP during hemiarch replacement is a safe alternative to using a sutured side graft. While cardiopulmonary bypass and circulatory arrest times appear improved, this is likely attributable to accumulation of experience and proficiency in technique. However, direct innominate artery cannulation may facilitate quicker completion of these procedures by eliminating the time necessary to suture a graft to the innominate artery.

## Introduction

Operations involving the ascending aorta and proximal aortic arch often utilize circulatory arrest to facilitate optimal surgical resection and reconstruction. However, cessation of blood flow to the brain risks permanent neurologic injury. 1 2 Strategies to improve neurologic outcomes during circulatory arrest include hypothermia often combined with retrograde or antegrade cerebral perfusion. Selective antegrade cerebral perfusion (SACP) has become the preferred method for cerebral protection during circulatory arrest at our institution. 3 4 5 Cannulation strategies for delivering SACP have evolved in recent years, with innominate artery cannulation becoming an increasingly utilized technique. 6 7 8 9

We transitioned from axillary to innominate artery cannulation to avoid the additional time and potential issues related to creating a separate incision for axillary cannulation. During our axillary experience, we targeted cerebral flow rates of > 10 mL/kg/min as this was widely espoused by the literature. 10 11 12 13 As such, this method required flow rates approaching 1 to 1.5 L/min making smaller cannulas, like 9-Fr root vents, inadequate. Originally, cannulation of the innominate artery was achieved by sewing a side graft to the vessel, allowing arterial access for both cardiopulmonary bypass (CPB) and delivery of SACP. 7 14 Our access strategy has since evolved to central aortic cannulation with use of a larger percutaneous cannula in the innominate artery for administration of SACP. Cannulation for innominate artery SACP is performed via a Seldinger technique using a 12- or 14-Fr cannula, allowing for higher flow rates while eliminating the time required to sew on a graft. 15 16 We hypothesized that SACP delivered via direct innominate artery cannulation using a 12- or 14-Fr cannula results in equivalent outcomes to cases utilizing an innominate side graft.

## Materials and Methods

### Patients

This was a single-center, retrospective analysis of 211 adult patients who underwent elective hemiarch replacement between 2012 and 2020. In all cases, hypothermic circulatory arrest was used during the distal aortic anastomosis with administration of SACP via the innominate artery. Urgent and emergent cases were excluded. Innominate cannulation for SACP was performed through either direct cannulation of the artery using a percutaneous cannula or via a 10-mm side graft sutured to the artery. Preoperative variables included age, gender, race, body mass index, medical comorbidities, symptoms on presentation, redo sternotomy, and concomitant surgical procedures. Intraoperative and postoperative outcomes included CPB time, aortic cross-clamp time, circulatory arrest time, nadir bladder temperature, intraoperative transfusions, length of stay, neurologic outcomes, end-organ dysfunction, need for operative reexploration, and mortality.

### Operative Techniques

**Fig. 1:**
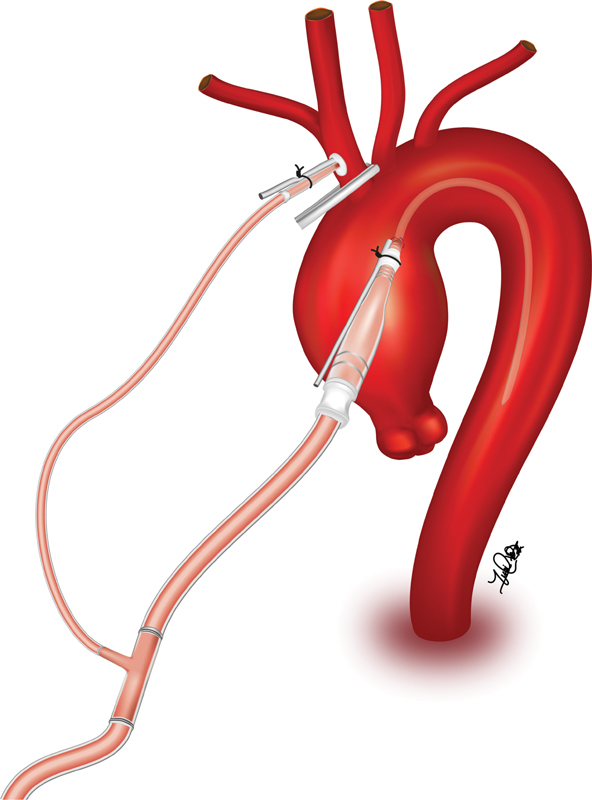
Central cannulation via the ascending aorta is utilized for establishment of cardiopulmonary bypass and initiation of cooling. Prior to circulatory arrest, the innominate artery is accessed with a needle and upsized to a 12- or 14-Fr cannula. Once the patient reaches the desired temperature, circulatory arrest ensues. The base of the innominate artery is occluded and selective antegrade cerebral perfusion is delivered via the 12- or 14-Fr cannula.

Elective hemiarch replacement cases included patients who required replacement of the ascending aorta with a single open distal anastomosis. Concomitant procedures were performed as indicated. For innominate grafting, a side biting clamp is used for proximal and distal control of the innominate artery. An arteriotomy is made and a 10-mm graft is sutured in an end-to-side fashion to the innominate artery using a 5–0 prolene suture. The innominate artery graft is then connected to the arterial line of the CPB circuit. For direct cannulation of the innominate artery, central cannulation via the ascending aorta is utilized for establishment of CPB followed by initiation of cooling ( Fig. 1 ). Prior to circulatory arrest, the innominate artery is accessed with a needle and upsized to a 12- or 14-Fr cannula depending on surgeon preference. Once the patient reaches the desired temperature, circulatory arrest ensues. The base of the innominate artery and left common carotid artery are occluded and SACP begins at 10 mL/kg/min via the side graft or percutaneous cannula, increasing flows as needed to preserve cerebral saturation bilaterally.

### Statistical Analysis

Bivariate analysis of preoperative variables and postoperative outcomes was conducted using the chi-square test and Fisher's exact test for categorical variables and the Student's *t* -test for continuous variables. Descriptive statistics are presented as absolute numbers and percentages for categorical variables. For continuous variables, statistics are presented as mean and standard deviation. A two-sided *p* -value of < 0.05 was considered statistically significant. All analyses were performed using GraphPad Prism 8 software (San Diego, CA). The study was approved by the Colorado Multiple Institutional Review Board. Individual patient consent was waived.

## Results

A total of 211 patients underwent elective hemiarch replacement with innominate artery cannulation and circulatory arrest with SACP during the study period. A side graft sutured to the innominate was utilized in 81% ( *N* = 171) of patients, while direct innominate artery cannulation was performed in 19% ( *N* = 40) of patients. Baseline patient characteristics are summarized in Table 1 . Characteristics were similar between groups aside from a higher baseline creatinine in the direct cannulation group (1.3 vs. 0.9, *p* = 0.032).

**Table 1:** Baseline characteristics of patients undergoing aortic hemiarch replacement using selective antegrade cerebral perfusion via a 10-mm side graft sutured to the innominate artery versus direct innominate cannulation using a 12- or 14-Fr cannula

	Side graft		Direct cannulation		*p* -Value
	Absolute value	Percentage	Absolute value	Percentage	
*N*	171		40		–
Age (y)	58.3 ± 15.0		59.9 ± 12.9		0.536
*Gender*					0.681
Male	130	76.0	32	80.0	
Female	41	24.0	8	20.0	
*Race/Ethnicity*					0.174
Caucasian	137	80.1	36	90.0	
Black/Other	34	19.9	4	10.0	
Body mass index	28.9 ± 6.3		29.1 ± 6.9		0.854
Baseline creatinine	0.9 ± 0.3		1.3 ± 1.8		**0.032**
Hypertension	110	64.3	25	62.5	0.856
Diabetes	19	11.1	8	20.0	0.185
Stroke	9	5.3	0	0.0	0.213
Chronic kidney disease	13	7.6	3	7.5	> 0.999
Coronary artery disease	26	15.2	4	10.0	0.463
Dyslipidemia	67	39.2	17	42.5	0.722
Pulmonary disease	31	18.1	9	22.5	0.508
Peripheral vascular disease	2	1.2	2	5.0	0.163
Smoking	58	33.9	11	27.5	0.575
Symptomatic on presentation	68	39.8	19	47.5	0.379
Redo sternotomy	20	11.7	4	10.0	> 0.999
*Concomitant procedures*					0.554
Aortic root replacement	71	41.5	18	45.0	
Isolated aortic valve replacement/repair	62	36.3	15	37.5	
Other a	35	20.5	5	12.5	

Note: Values are presented as mean ± standard deviation (SD) unless otherwise specified.

a Coronary artery bypass grafting, mitral valve repair, mitral valve replacement, patent foramen ovale closure, aortic annulus enlargement, and/or atrial fibrillation procedure.

Patients undergoing direct cannulation of the innominate artery demonstrated shorter CPB time (132.7 vs. 154.9 minutes, *p* = 0.020) and shorter circulatory arrest time (8.1 vs. 10.9 minutes, *p* = 0.004; Table 2 ). Nadir bladder temperature did not significantly differ between groups (27.2°C for side graft vs. 27.6°C for direct cannulation, *p* = 0.088). There were no significant differences in postoperative outcomes including intraoperative transfusions, length of stay, stroke, delirium, renal failure requiring renal replacement therapy, myocardial infarction, pneumonia, respiratory failure requiring mechanical ventilation > 48 hours, operative reexploration, and in-hospital death.

**Table 2:** Intraoperative and postoperative outcomes following elective aortic hemiarch replacement using selective antegrade cerebral perfusion via a 10-mm side graft sutured to the innominate artery versus direct innominate cannulation using a 12- or 14-Fr cannula

	Side graft		Direct cannulation		*p* -Value
	Absolute value	Percentage	Absolute value	Percentage	
*N*	171		40		–
Cardiopulmonary bypass time (min)	154.9 ± 56.2		132.7 ± 40.7		**0.020**
Cross-clamp time (min)	104.8 ± 51.7		93.9 ± 34.1		0.202
Circulatory arrest time (min)	10.9 ± 6.0		8.1 ± 1.9		**0.004**
Nadir bladder temperature (°C)	27.2 ± 1.2		27.6 ± 0.7		0.088
*Intraoperative transfusion products*					
Total	4.7 ± 6.8		3.5 ± 3.5		0.269
Packed red blood cells	0.7 ± 2.4		0.6 ± 1.3		0.771
Fresh-frozen plasma	2.7 ± 3.8		1.8 ± 1.9		0.146
Platelets	1.3 ± 1.2		1.1 ± 1.0		0.258
Length of stay (d)	7.3 ± 7.9		8.3 ± 4.6		0.456
Stroke	4	2.3	1	2.5	> 0.999
Delirium	9	5.3	5	12.5	0.149
Renal failure	2	1.2	1	2.5	0.470
Myocardial infarction	0	0.0	0	0.0	> 0.999
Pneumonia	3	1.8	1	2.5	0.572
Respiratory failure	4	2.3	2	5.0	0.319
Operative re-exploration	11	6.4	3	7.5	0.732
Postoperative death	2	1.2	1	2.5	0.470

Note: Values are presented as mean ± standard deviation (SD) unless otherwise specified.

## Discussion

Strategies for administration of SACP during circulatory arrest for open aortic arch cases are continuously evolving with ever improving neurologic outcomes. Historically, axillary artery cannulation welcomed the age of SACP. 17 18 19 However, this technique can be time consuming, introduces a disparate site of bleeding throughout the case, and leads to potential complications such as nerve injury, limb malperfusion, and seroma. 20 Cannulation of the innominate artery has since been shown to be a safe and effective alternative to axillary cannulation, avoiding the need for a second incision and providing a larger caliber vessel for systemic and cerebral flow such that a separate site of arterial cannulation is not needed. 6 21 The most widely accepted approach to innominate cannulation is via a side graft anastomosed to the innominate artery. 6 7 22 23 While direct cannulation of the innominate artery for SACP has become an increasingly utilized technique, the limited flow rates achievable through smaller cannulas did not support our approach to cerebral perfusion. Moreover, with experience, we aimed to transition to a more efficient form of cannulation in an effort to obviate delays in the initiation of cooling. This strategy, termed “turbocooling,” uses central cannulation of the ascending aorta for CPB combined with direct cannulation of the innominate artery for SACP using a 12- or 14-Fr cannula, thus enabling us to utilize the excess bypass time required for cooling and warming instead of adding more time to the case overall. The results of this study indicate that direct cannulation of the innominate artery for SACP with a 12- or 14-Fr cannula is a safe alternative to using a sutured side graft without compromising our cerebral flow protocol. Compared with a side graft, this strategy demonstrates equivalent neurologic outcomes and rates of end-organ dysfunction. While CPB and circulatory arrest times appear reduced with direct cannulation, this is more likely attributable to evolution of the operation rather than a reflection of cannulation strategy alone.

Previously described reports on direct innominate cannulation utilize a variety of cannulation sizes. Our choice of a 12- or 14-Fr cannula mitigates the risks associated with larger cannulas while preserving adequate perfusion pressure and flow that is often not achievable with smaller cannulas. Studies describing innominate artery cannulation using large 20- to 24-Fr cannulas allow for establishment of CPB in addition to delivery of SACP. 21 24 25 However, these larger caliber cannulas risk vessel injury, particularly if the innominate artery is small, and require manipulation of the cannula multiple times throughout the case to transition between systemic perfusion and cerebral perfusion, further increasing the potential for vascular injury and creation of emboli. In contrast, the use of small cannulas for delivery of SACP has also been described. Jassar et al 15 reported their experience in 100 patients undergoing elective hemiarch replacement using a 9-Fr catheter in the innominate artery for SACP with postoperative neurologic events occurring in 2% of patients. Similarly, a study by Payabyab et al 16 describe the use of a 7-Fr aortic root cannula in the innominate artery for administration of SACP in patients presenting with Type A aortic dissection.

The use of a 12- to 14-Fr cannula as described in this study reduces the risk of vascular injury while maintaining optimal perfusion to the brain by achieving flow rates of up to 1 to 1.5 L/min. Although a smaller diameter cannula is theoretically less traumatic, it may impede adequate cerebral perfusion pressure and flow, which is of primary importance for attaining cerebral protection during circulatory arrest and preventing devastating neurologic complications. The slightly larger cannulas used at our institution are more likely to guarantee consistent cerebral perfusion in all patients. In the only other published study using a 14-Fr cannula for direct innominate cannulation for delivery of SACP, Garg et al 8 report comparable outcomes to axillary artery cannulation with a 2% stroke and 2% mortality rate. Direct cannulation of the innominate avoids the need for partial or complete occlusion of the artery, as well as eliminates the time required to sew a side graft to the artery. While use of a side graft provides a single arterial cannulation site for both CPB and SACP, the addition of a separate cannulation site for SACP with central aortic cannulation is still quick to perform and requires only one additional suture. It thus remains a practical and efficient option as a cannulation strategy for many cardiac surgeons.

There are several limitations of this study that warrant mention. First, the small case number and single-institution design limit the generalizability of the results. Additionally, our findings must be interpreted with caution due to the methodological limitations associated with the retrospective, observational design of this study. This cohort includes only elective cases, limiting the applicability of these results to urgent or emergent hemiarch replacements. We recognize the presence of temporal bias in regard to the two techniques, with the majority of direct cannulation cases occurring within the most recent 3 years of the 8-year study period. While CPB and circulatory arrest times appear improved for patients undergoing direct cannulation, this is likely a reflection of accumulated experience and proficiency in technique rather than cannulation strategy alone. Information regarding neurologic outcomes and functional status posthospitalization is an important adjunct for assessing long-term morbidity; however, this was difficult to consistently ascertain from the medical charts, and thus was not included as an outcome variable.

## Conclusion

Outcomes following direct innominate artery cannulation with a 12- or 14-Fr cannula for SACP during elective hemiarch replacement are comparable to using a sutured side graft. This is therefore a safe alternative to the current accepted technique for administration of SACP via the innominate artery while allowing for improved cerebral flow compared with previously described smaller percutaneous cannulas. Direct cannulation may facilitate quicker completion of these procedures by eliminating the time necessary to suture a graft to the innominate artery; however, prospective, large-scale studies are needed to adequately evaluate these potential advantages.
